# Insights on diverse roles of glutathione metabolism in regulating the browning of fresh-cut produce

**DOI:** 10.3389/fnut.2026.1998961

**Published:** 2026-10-29

**Authors:** Siqi Xu, Xiao Yuan, Qingli Wang, Yuanyuan Jiang, Bin Wang

**Affiliations:** 1 College of Biology and Agriculture, Shaoguan University, Shaoguan, China; 2 Guangdong Provincial Key Laboratory of Utilization and Conservation of Food and Medicinal Resources in Northern Region, Shaoguan University, Shaoguan, China; 3 Guangdong Provincial Engineering and Technology Research Center of Special Fruit and Vegetables in Northern Region, Shaoguan University, Shaoguan, China

**Keywords:** fresh-cut produce, fruit and vegetable, non-enzymatic antioxidants, ROS balance, surface browning

## Abstract

The postharvest preservation of fresh-cut produce remains a major challenge, largely due to wound-induced enzymatic browning. Historically, research in this area has focused primarily on oxidase-mediated pathways. Glutathione (GSH), a non-protein tripeptide thiol widely distributed across the plant kingdom, has traditionally been viewed merely as a sacrificial chemical antioxidant that mitigates browning. In this mini-review, we discuss the multi-tiered defense system governed by GSH metabolism in regulating the browning of fresh-cut produce. Based on existing evidence, we propose that GSH is not merely a passive reactive oxygen species scavenger. Instead, it may serve as a homeostatic orchestrator through multiple layers of regulation, including PPO inhibition, redox signaling, energy metabolism, and secondary metabolite allocation. Due to limited literature regarding GSH metabolism in fresh-cut produce, some of these intricate mechanisms are currently inferred from model plant systems, such as *Arabidopsis thaliana*. More direct evidence is required in diverse fresh-cut horticultural products. In all, a deeper understanding of these multifaceted roles of GSH metabolism in enzymatic browning will provide fresh insights into the development of targeted anti-browning strategies in the fresh-cut industry.

## 1 Introduction

Surface browning is one of the major quality deterioration issues limiting the shelf life and consumer acceptance of fresh-cut fruits and vegetables (1). It is widely established that this browning is enzymatic, driven primarily by oxidative enzymes such as polyphenol oxidase (PPO) and peroxidase (POD) (2, 3). To date, most reviews have focused on oxidative enzymes-centered mechanisms, often isolating this discoloration as a terminal biochemical event occurring strictly at the cut surface (4–6). However, mitigating postharvest browning in minimally processed commodities requires a holistic perspective, one that transcends the downstream inhibition of specific enzymes. Emerging paradigms in plant physiology underscore that browning kinetics are intrinsically intertwined with global cellular redox status, wound-induced stress signaling, and localized energetic homeostasis (7).

Glutathione (GSH), frequently designated as the “master antioxidant” or “cellular defender,” is a non-protein tripeptide thiol widely distributed across the plant kingdom (8). Accumulating evidence reveals that GSH metabolism is intimately linked to the browning of fresh-cut produce (9), implying that its diverse physiological roles are deeply integrated into this process. However, conventional postharvest research predominantly confines the utility of GSH to that of a passive chemical additive or a straightforward reactive oxygen species (ROS) scavenger within the ascorbate-glutathione (AsA-GSH) cycle (10, 11). This narrow perspective significantly underestimates the physiological complexity of GSH, leaving a distinct gap between practical biochemical applications and molecular insights within the fresh-cut sector.

To bridge this gap, this mini-review aims to move beyond the reductionist view of GSH as merely an antioxidant additive. In this mini-review, we summarize and discuss the multifaceted roles of GSH metabolism in plants and further discuss how these functions may contribute to the regulation of browning in fresh-cut fruits and vegetables. Ultimately, this review seeks to provide new perspectives for the development of targeted, next-generation preservation strategies.

## 2 Technologies can modulate GSH metabolism to alleviate fresh-cut browning

To date, an array of postharvest preservation technologies has been investigated to control tissue discoloration in fresh produce, particularly fresh-cut fruit and vegetables, by regulating GSH metabolism (Table 1). These strategies encompass both the direct application of exogenous GSH and alternative interventions designed to target or modulate endogenous GSH pathways, thereby orchestrating a comprehensive defense system against enzymatic browning.

**Table 1:** Postharvest technologies to alleviate tissue browning in postharvest produce by modulating GSH metabolism.

Species	Anti-browning agent or technology	Major contribution	Reference
Lotus	GSH	Enhancing PAL, APX, GR, catalase and SOD activities, suppressing PPO and POD activities, elevating AsA, GSH and GSSG contents	(10)
Lotus	Ultrasonic combined with GSH	Improving GSH efficiency, enhancing ROS scavenging activity	(12)
Apple	Protocatechualdehyde	Regulating phenolic metabolism, reducing membrane lipid peroxidation, enhancing the AsA-GSH cycle, and modulating cell wall metabolism	(13)
Taro	Vanillic acid and cinnamic acid	Boosting GSH metabolism	(11, 14)
Potato	AsA	Regulating GSH metabolism, enhancing starch metabolism, and inhibiting phenylalanine metabolism, sucrose degradation, and fatty acid synthesis	(15)
Chinese water chestnut	Hydrogen sulfide	Enhancing the activities of APX and GR	(16)
Cornelian cherry	Sodium nitroprusside and γ-aminobutyric acid	Upregulating GR and related antioxidant enzyme activities	(17)
Pitaya	Hot air	Activating the AsA-GSH cycle	(18)
Potato	Vacuum combined with ascorbic acid	Activating GSH-mediated antioxidant pathways	(19)

### 2.1 Direct application of GSH

Some studies have validated that the direct exogenous application of GSH, *via* dipping, spraying, or vacuum infiltration, can effectively mitigate surface browning in several fresh-cut horticultural species. In fresh-cut lotus root slices, a 1% (w/v) exogenous GSH application significantly delayed surface discoloration during cold storage by suppressing the activities of browning-related enzymes while concurrently preserving higher internal ascorbic acid (AsA) levels (10). A similar anti-browning efficacy was demonstrated in fresh-cut wax apple fruit, where a 1% (w/v) GSH dipping treatment markedly reduced the incidence of surface browning and retarded the decline of tissue lightness under refrigerated storage conditions (20). Furthermore, combining exogenous GSH with physical processing technologies yields synergistic benefits, enhancing the overall efficacy of browning mitigation. For instance, treating fresh-cut lotus rhizome slices with 40-kHz ultrasonication (US) for 2 min in tandem with a lower concentration of GSH (0.5%, w/v) inhibited cut-surface browning far more effectively than either the GSH or US treatment alone (12).

### 2.2 Indirect modulation of GSH metabolism

#### 2.2.1 Chemical elicitors or natural compounds other than GSH

The application of exogenous chemical elicitors and natural bioactive compounds significantly regulates the activities of browning-related enzymes and strengthens the AsA-GSH cycle to efficiently quench wound-induced ROS generation. For instance, vanillic acid and cinnamic acid, two prominent phenolic acids, effectively mitigate tissue discoloration in taro slices by partially boosting GSH metabolism (11, 14). Similarly, AsA has been shown to modulate the physiological responses to wound healing in fresh-cut potatoes; it achieves this by regulating GSH and starch metabolism while simultaneously suppressing phenylalanine metabolism, sucrose degradation, and fatty acid synthesis (15). Moreover, natural phenolic compounds such as protocatechualdehyde can markedly enhance postharvest quality by indirectly bolstering the enzymatic components that drive the AsA-GSH cycle (13).

#### 2.2.2 Gaseous signaling treatments

Gaseous fumigation technologies have emerged as efficient, non-destructive tools that interface deeply with plant stress signaling networks and GSH homeostasis. For instance, hydrogen sulfide (H_2_S) fumigation enhances browning repression through the sustained activation of the enzymatic antioxidant defense system, particularly by elevating ascorbate peroxidase (APX) and glutathione reductase (GR) activities (16). Similarly, exogenous nitric oxide (NO) suppresses tissue discoloration by upregulating the activities of GR and related antioxidant enzymes in horticultural crops. For example, in whole cornelian cherry fruit, treatment with sodium nitroprusside (a NO donor) preserves membrane integrity and prevents fruit browning by modulating the activities of enzymes involved in GSH metabolism, thereby restricting the accumulation of ROS, such as hydrogen peroxide (H₂O₂) and superoxide radicals ( $O_{2}^{\cdot -}$ ), as well as malondialdehyde (17).

#### 2.2.3 Physical technologies

Physical therapies reshape GSH-mediated metabolic pathways by acting as beneficial environmental stresses. For instance, pre-treating fresh-cut pitaya fruit with hot air at 42 °C for 3 h effectively alleviates tissue browning during subsequent storage at both 4 °C for 14 d and 20 °C for 48 h, a protective effect primarily driven by the robust activation of the AsA-GSH cycle (18). Moreover, combining physical interventions with biochemical agents can yield pronounced synergistic effects. A prominent example is the integration of vacuum packaging with exogenous AsA treatment, which markedly mitigates the surface browning of fresh-cut potato slices by significantly enhancing endogenous GSH utilization efficiency and stabilizing cellular redox status (19).

These studies demonstrate that the direct application of GSH or other technologies can successfully inhibit browning in fresh-cut produce, either directly or indirectly through GSH-mediated functions. However, they predominantly restrict their focus to downstream antioxidant responses, such as ROS scavenging or direct enzyme inhibition. This reductionist perspective treats GSH merely as a superficial chemical additive or a static biochemical sink. Consequently, it fails to answer the deep-seated physiological mechanisms, thereby limiting the consideration of GSH metabolism as a target for browning control.

## 3 Possible mechanisms of GSH metabolism involving in browning control

Undoubtedly, the primary mechanism by which GSH regulates browning is attributed to its direct antioxidant protectiveness. However, to conceptualize GSH exclusively as a passive chemical scavenger or a downstream antioxidant buffer represents an incomplete physiological narrative. Beyond its direct antioxidant activity, emerging evidence suggests that GSH may contribute to the mitigation of fresh-cut browning through broader regulatory processes involving redox signaling, metabolic adjustment, and cellular homeostasis.

### 3.1 Direct antioxidant effects

At the physiological level, cutting-induced mechanical wounding triggers an immediate burst of ROS, primarily consisting of $O_{2}^{\cdot -}$ and H₂O₂ (21, 22). Subsequently, $O_{2}^{\cdot -}$ is then dismutated into more stable and soluble H₂O₂ molecule, a process catalyzed by superoxide dismutase (SOD) (23). These accumulated ROS directly attack cellular biomacromolecules, particularly membrane lipids, inducing severe lipid peroxidation that ultimately compromises cellular compartmentalization and elevates membrane permeability (24, 25). This structural disruption allows PPO to physically mix with its phenolic substrates, accelerating the enzymatic synthesis of *o*-quinones.

In botanical systems, GSH counteracts this cascade as a cornerstone non-enzymatic antioxidant, both through maintaining cellular redox homeostasis by the AsA-GSH cycle-mediated ROS detoxification (26). Redox-sensitive green fluorescent protein (roGFP)-based studies in plants have demonstrated that GSH-dependent redox regulation is highly compartment-specific, suggesting a potential role of subcellular redox homeostasis in maintaining membrane integrity during wounding stress (27). Sustained by NADPH-dependent GR, a high GSH/GSSG ratio pools reducing equivalents into the AsA-GSH cycle, driving APX to scavenge localized H₂O₂ (28). Crucially, this localized redox buffering suppresses the lipid peroxidation detailed above, thereby safeguarding the integrity of the tonoplast envelopes. By preserving these membrane barriers, the AsA-GSH cycle may help maintain cellular compartmentation between PPO and phenolic substrates (such as chlorogenic acid and catechin), thereby limiting the initiation and progression of enzymatic browning.

Undeniably, the direct antioxidant effects of GSH are crucial for suppressing browning through scavenging ROS in fresh-cut produce. However, to limit its action to radical interception overlooks its diverse roles in regulating enzymatic browning.

### 3.2 Direct or indirect inhibition of PPO activity

Beyond buffering upstream oxidative stress, GSH possibly intervenes in the downstream catalytic machinery of enzymatic browning through a dual-action mechanism involving active-site coordination and quinone interception. Mechanistic kinetic studies reveal that GSH can act as a mixed-type or non-competitive inhibitor of plant PPOs, especially apple fruit (29, 30). The sulfhydryl (-SH) group of GSH may contribute to the inhibition of PPO; however, the specific mechanisms are primarily interpreted from model study. Studies using purified apple PPO have shown that GSH can react with enzyme-generated *o*-quinones to form colorless conjugates, thereby limiting pigment formation. However, the observed inhibition of PPO activity by GSH was largely attributable to the decrease in reaction pH under the conditions tested (31). By comparison, studies of L-cysteine-mediated PPO inhibition have suggested that thiol-containing compounds may inhibit browning through interactions with enzyme-generated quinones and, potentially, with the enzyme itself (32).

Concurrently, if *o*-quinones are inevitably synthesized, GSH potentially interrupts their subsequent non-enzymatic polymerization into brown melanins. Via a rapid nucleophilic addition reaction, the thiol group of GSH attacks the electrophilic aromatic ring of highly reactive *o*-quinones. This process yields stable, colorless thiol-conjugates, commonly referred to as GSH-phenolic adducts, such as 2-*S*-glutathionyl chlorogenic acid (33, 34). Because these thiol-conjugates possess altered steric and electronic properties, they may fail to serve as substrates for further PPO-mediated oxidation (35). These conjugates may reduce the availability of reactive quinones for polymerization and therefore limit pigment formation.

### 3.3 Intracellular signaling transduction and gene expression

At the molecular level, the GSH/GSSG pool may function as an important component of cellular redox signaling that translates cellular redox fluctuations into nuclear transcriptional shifts in plants (36–38). Mechanical injury causes a transient depletion of reduced GSH and an accumulation of GSSG (oxidized GSH), shifting the cellular redox potential toward an oxidative state. This redox flux triggers a post-translational modification known as protein *S*-glutathionylation, where specific, solvent-accessible cysteine residues on target proteins form reversible mixed disulfide bonds with GSH (39, 40). In plant stress responses, *S*-glutathionylation alters the activity and conformation of key metabolic enzymes and transcription factor (TF) complexes, preventing their irreversible over-oxidation while fine-tuning downstream signal transduction.

Simultaneously, GSH may interact with hormone-dependent signaling networks involved in wound responses, thereby influencing the expression of genes associated with phenylpropanoid metabolism and oxidative defense. In plant, wounding stress typically induces the accumulation of several key phytohormones such as jasmonic acid (JA) and ethylene (ETH), which upregulate the transcription of key genes in the phenylpropanoid pathway, such as phenylalanine ammonia-lyase (PAL), and cinnamate 4-hydroxylase, as well as phenoloxidase, such as PPO (41–43). Exogenous or elevated endogenous GSH dynamically regulates the transcript abundance of these biosynthetic or catalytic genes (10, 44). This transcriptional regulation may be mediated by redox-sensitive TF networks, such as specific WRKY and MYB families, which further dampen the synthesis of both browning enzymes and their phenolic substrates (45).

However, it should emphasize that these transcriptional networks and hormonal cross-talk dynamics have been mapped primarily in model plant systems such as *Arabidopsis thaliana*. Direct empirical validation of these specific redox-sensitive TF switches remains limited in fresh-cut horticultural commodities.

### 3.4 Metabolic allocation of energy and secondary metabolites

The spatial containment of browning may be an energy-dependent process integrated with vacuolar metabolic flux allocation. In plants, maintaining the structural segregation between plastidic PPO and vacuolar phenolics may require a supply of adenosine triphosphate (ATP) to sustain the electrochemical gradient across the tonoplast (46). This process is primarily powered by vacuolar H^+^-ATPase and H^+^-pyrophosphatase pumps in plants (47, 48). Under the energy crisis induced by fresh-cut processing, ATP depletion could compromise these proton pumps, potentially leading to membrane leakage and substrate mixing. Within this context, GSH is hypothesized to support mitochondrial structural integrity by scavenging electron transport chain-derived ROS, which may help preserve mitochondrial function.

Furthermore, GSH metabolism may also direct the metabolic flux of secondary metabolites away from the cytosolic compartment where active PPO resides. Certain plant glutathione transferases (GSTs), particularly members of the Phi and Tau classes, possess ligand-binding properties and may participate in the intracellular trafficking or sequestration of specific secondary metabolites (49). Rather than catabolizing these compounds, GSTs may facilitate their intracellular escort to the tonoplast. ATP-binding cassette (ABC) transporters utilize energy from ATP hydrolysis to pump GSH conjugates or phenolic-GSH adducts into the vacuolar lumen (50, 51). By driving this GST-mediated vacuolar sequestration, a sustained GSH metabolic pool ensures the clearance of highly reactive phenolic substrates from the cytosol, minimizing the risk of localized browning even if minor membrane leakage occurs.

While this GST-mediated vacuolar sequestration model is characterized in model plants, direct evidence confirming that fresh-cut tissue relies on the identical transport systems for browning mitigation is still sparse. Consequently, caution should be exercised when extrapolating these precise intracellular trafficking mechanisms to diverse horticultural crops without further crop-specific empirical data.

## 4 Conclusion

A comprehensive schematic summarizing these mechanisms of GSH-mediated browning regulation is presented in Figure 1. In conclusion, far beyond its classical role as a sacrificial chemical scavenger, GSH may operate as a proactive homeostatic orchestrator in fresh-cut tissues possibly through a multi-tiered defense system. While the direct scavenging of wound-induced ROS *via* the AsA-GSH cycle and the biochemical inactivation of PPO through copper chelation or thiol-quinone conjugation are well-supported by direct evidence in horticultural produce, other upstream and downstream regulatory arms require further validation. Specifically, mechanisms involving the modulation of nuclear transcriptional shifts *via S*-glutathionylation, the preservation of mitochondrial energy charge, and the GST-mediated vacuolar sequestration of secondary metabolites remain largely predictive, being inferred from model plant systems. Future research should bridge these gaps by transitioning from model-plant paradigms to crop-specific functional validation, thereby transforming these mechanistic deductions into concrete, translatable anti-browning technologies for the fresh-cut industry.

**Figure 1:**
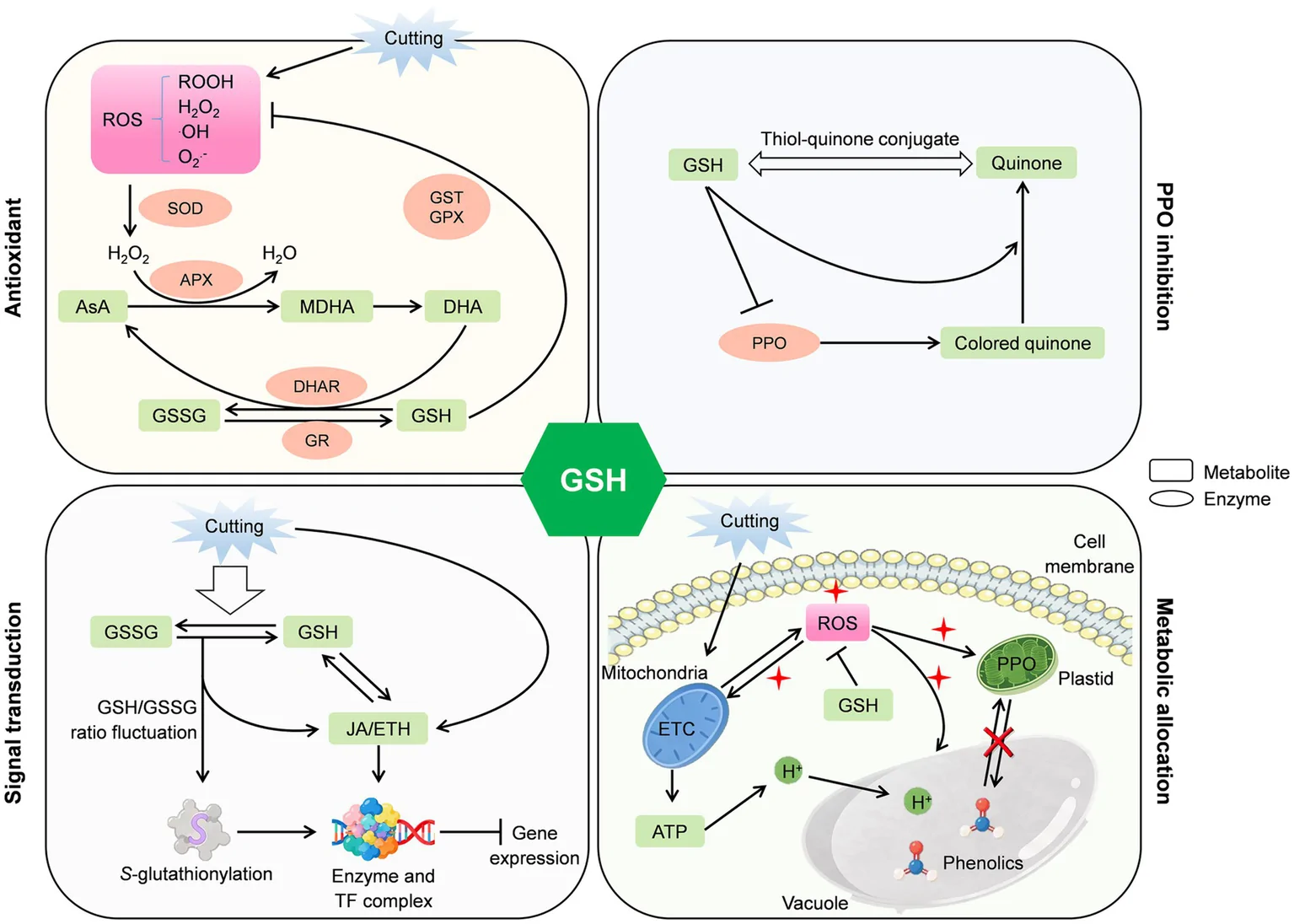
A schematic overview of the multi-tiered defense mechanisms mediated by glutathione (GSH) in regulating enzymatic browning and oxidative stress in fresh-cut horticultural products. ‘→’ indicates a promotive effect, while ‘⊣’ signifies an inhibitory effect. The abbreviations can be found in the main text. Elements of cell membrane were adapted from Servier Medical Art, provided by Servier, licensed under CC BY 3.0. Other cellular components were generated by the DuMate AI tool.

## 5 Perspective

Despite the above mechanistic insights, several critical knowledge gaps remain, highlighting prominent directions for future research:

(1) Current understanding of the spatiotemporal dynamics of organelle-specific GSH pools relies heavily on bulk tissue analysis, which masks localized cellular responses. Future investigations should utilize advanced, genetically encoded biosensors (e.g., roGFP) to track real-time, organelle-specific (mitochondrial, plastidial, and cytosolic) GSH/GSSG fluctuations during the immediate post-wounding phases, thereby clarifying how redox shifts command the earliest browning signals.
(2) While the expression downregulation of browning-related genes such as *PPO* or *PAL* mediated by GSH is documented, the exact upstream TFs and phytohormones targeted by GSH-driven redox signaling remain to be fully elucidated.
(3) Currently, the direct application of exogenous GSH to control browning in fresh-cut produce remains challenging because of concerns regarding cost-in-use, potential sensory effects (e.g., sulfurous off-odors and bitter notes), residue management, and regulatory compliance. Future research should focus on improving the efficacy of exogenous GSH application, potentially through synergistic combinations with physical treatments, such as ultrasound, to achieve effective browning control across diverse fresh-cut commodities. Alternatively, modulating endogenous GSH metabolism through chemical or physical treatments may offer a promising strategy for controlling browning in fresh-cut produce.

To systematically resolve these interconnected bottlenecks, integrating multi-omics approaches, particularly redox-proteomics, spatiotemporal metabolomics, and single-cell transcriptomic, may provide a holistic, multi-layered blueprint of the GSH-mediated regulatory network. This strategy could map dynamic redox modifications of core signaling proteins, capture real-time metabolic flux rerouting, and pinpoint the missing upstream TFs and phytohormones at a system-wide scale. Such integrated omics frameworks have already proven powerful in unravelling the complex regulatory cascades that govern postharvest browning in fresh-cut produce. Ultimately, these insights will in turn pave the way for innovative, targeted postharvest technologies designed to extend shelf-life and preserve the nutritional value of fresh-cut produce.
